# Individuals with presumably hereditary uveal melanoma do not harbour germline mutations in the coding regions of either the P16^INK4A^, P14^ARF^ or cdk4 genes

**DOI:** 10.1054/bjoc.1999.1005

**Published:** 2000-02

**Authors:** N Soufir, B Bressac-de Paillerets, L Desjardins, C Lévy, J Bombled, I Gorin, P Schlienger, D Stoppa-Lyonnet

**Affiliations:** Unité des Marqueurs Génétiques des Cancers, Institut Gustave Roussy, 45 rue Camilles Desmoulins, Villejuif, F94805 cedex, France; Service d'Oncologie Ophtalmologique, Consultation de Dermatologie, Service de Radiothérapie C, Service de Génétique Oncologique, Institut Curie, 26 rue d'Ulm, Paris, F75231, France

**Keywords:** uveal melanoma, germline mutation, P16^INK4A^, P14^ARF^, cdk4

## Abstract

In familial cutaneous malignant melanoma (CMM), disruption of the retinoblastoma (pRB) pathway frequently occurs through inactivating mutations in the *p16 (p16^INK4A^/CDKN2A/MTS1*) gene or activating mutations in the G1-specific cyclin dependent kinase 4 gene (*CDK4*). Uveal malignant melanoma (UMM) also occurs in a familial setting, or sometimes in association with familial or sporadic CMM. Molecular studies of sporadic UMM have revealed somatic deletions covering the INK4A-ARF locus (encoding P16^INK4A^and P14^ARF^) in a large proportion of tumours. We hypothesized that germline mutations in the *p16^INK4A^*, *p14^ARF^*or *CDK4* genes might contribute to some cases of familial UMM, or to some cases of UMM associated with another melanoma. Out of 155 patients treated at the Institut Curie for UMM between 1994 and 1997, and interviewed about their personal and familial history of melanoma, we identified seven patients with a relative affected with UMM (*n* = 6) or CMM (*n* = 1), and two patients who have had, in addition to UMM, a personal history of second melanoma, UMM (*n* = 1), or CMM (*n* = 1). We screened by polymerase chain reaction single-strand conformation polymorphism the entire coding sequence of the INK4A-ARF locus (exon 1α from *p16^INK4A^*, exon 1β from *p14^ARF^*, and exons 2 and 3, common to both genes), as well as the exons 2, 5 and 8 of the *CDK4* gene, coding for the functional domains involved in p16 and/or cyclin D1 binding. A previously reported polymorphism in exon 3 of the INK4A-ARF locus was found in one patient affected with bilateral UMM, but no germline mutations were detected, either in the *p16^INK4A^*, *p14^ARF^*or *CDK4* genes. Our data support the involvement of other genes in predisposition to uveal melanoma. © 2000 Cancer Research Campaign


British Journal of Cancer (2000) **82**(4), 818–822
© 2000 Cancer Research Campaign
DOI: 10.1054/ bjoc.1999.1005, available online at http://www.idealibrary.com on IDEAL®

### Individuals with presumably hereditary uveal melanoma do not harbour germline mutations in the coding regions of either the P16$^{INK4A}$, P14$^{ARF}$ or cdk4 genes

**N Soufir[1,*], B Bressac-de Paillerets[1], L Desjardins[2], C Lévy[2], J Bombled[1], I Gorin[3], P Schlienger[4] and D Stoppa-Lyonnet[5]**

[1]Unité des Marqueurs Génétiques des Cancers, Institut Gustave Roussy, 45 rue Camilles Desmoulins, Villejuif, F94805 cedex, France, [2]Service d'Oncologie Ophtalmologique, [3]Consultation de Dermatologie, [4]Service de Radiothérapie C, [5]Service de Génétique Oncologique, Institut Curie, 26 rue d'Ulm, Paris, F75231, France

**Summary** In familial cutaneous malignant melanoma (CMM), disruption of the retinoblastoma (pRB) pathway frequently occurs through inactivating mutations in the *p16 (p16$^{INK4A}$/CDKN2A/MTS1*) gene or activating mutations in the G1-specific cyclin dependent kinase 4 gene (*CDK4*). Uveal malignant melanoma (UMM) also occurs in a familial setting, or sometimes in association with familial or sporadic CMM. Molecular studies of sporadic UMM have revealed somatic deletions covering the INK4A-ARF locus (encoding P16$^{INK4A}$ and P14$^{ARF}$) in a large proportion of tumours. We hypothesized that germline mutations in the *p16$^{INK4A}$*, *p14$^{ARF}$* or *CDK4* genes might contribute to some cases of familial UMM, or to some cases of UMM associated with another melanoma. Out of 155 patients treated at the Institut Curie for UMM between 1994 and 1997, and interviewed about their personal and familial history of melanoma, we identified seven patients with a relative affected with UMM ($n$ = 6) or CMM ($n$ = 1), and two patients who have had, in addition to UMM, a personal history of second melanoma, UMM ($n$ = 1), or CMM ($n$ = 1). We screened by polymerase chain reaction single-strand conformation polymorphism the entire coding sequence of the INK4A-ARF locus (exon 1α from *p16$^{INK4A}$*, exon 1β from *p14$^{ARF}$*, and exons 2 and 3, common to both genes), as well as the exons 2, 5 and 8 of the *CDK4* gene, coding for the functional domains involved in p16 and/or cyclin D1 binding. A previously reported polymorphism in exon 3 of the INK4A-ARF locus was found in one patient affected with bilateral UMM, but no germline mutations were detected, either in the *p16$^{INK4A}$*, *p14$^{ARF}$* or *CDK4* genes. Our data support the involvement of other genes in predisposition to uveal melanoma. © 2000 Cancer Research Campaign

**Keywords**: uveal melanoma; germline mutation; P16$^{INK4A}$; P14$^{ARF}$; cdk4

Uveal malignant melanoma (UMM) is a rare malignant adult neoplasm (incidence: 1/1 000 000), but it is the most common intraocular primary malignancy. UMM can occur in a familial setting (reviewed in Canning and Hungerford, 1988). Large studies have statistically demonstrated an excess of UMM risk in relatives of UMM patients (Singh et al, 1996*b*) and it is now assumed that at least 0.6% of all uveal melanoma cases are familial (Singh et al, 1996*a*). Moreover, observation of large pedigrees has shown that transmission was autosomal dominant with incomplete penetrance (Lynch et al, 1968), and that bilateral uveal melanoma occurred more frequently than expected (Singh et al, 1996*e*). Numerous clinical and biological data suggest common hereditary factors for UMM and cutaneous melanoma (CMM). UMM may occur in familial CMM probands (Newton Bishop et al, 1994; Van Hees et al, 1998). CMM may be present in first-degree relatives of UMM patients, often in association with dysplastic naevus syndrome (DNS) (Van Hees et al, 1998). Co-existence of UMM and CMM in a same individual occur in up to 2% of UMM patients (Bataille et al, 1993; Van Hees et al, 1998). Finally, uveal and cutaneous melanocytes share a common embryology, originating in the neural crest. Both cells migrate to their respective site during embryological development, and may give rise to naevi and in some instances, to melanomas.

Some other clinical conditions seem to predispose to UMM: ocular melanocytosis, neurofibromatosis type I, and Li–Fraumeni syndrome (Singh et al, 1996*e*). At a genetic level, BRCA2 germline mutations may be associated with an increased risk of UMM (Easton et al, 1997; Sinilnikova et al, 1999).

Inactivating germline mutations of the *p16$^{INK4A}$* gene have been found to segregate with the disease in 15–50% of CMM families (familial atypical mole and melanoma syndrome (FAMMM) (reviewed in Dracopoli and Fountain, 1996). The INK4A-ARF locus localized at 9p21 encodes two alternative reading frame proteins, P16$^{INK4A}$ (exons 1α, 2 and 3) and P14$^{ARF}$ (exons 1β, 2 and 3), both involved in the negative control of cell proliferation. P16$^{INK4A}$ produces a G1 cell cycle arrest by inhibiting phosphorylation of the retinoblastoma protein by the cyclin-dependent kinases cdk4 and cdk6 (Serrano et al, 1993). P14$^{ARF}$ is a structurally different protein, which has been recently shown to act both at G1/S and G2/M phases, in a p53-dependent manner. This is done via binding and inhibition of the protein MDM2, which itself promotes P53 degradation (Stott et al, 1998). In addition, its murine homologue, P19$^{ARF}$, can directly associate with P53 (Kamijo et al, 1998).

*Received 2 June 1999*
*Revised 27 September 1999*
*Accepted 28 September 1999*

*Correspondence to*: D Stoppa-Lyonnet

*Present address: Institut de Recherche Sur la Peau, Inserm U312, Hôpital Saint-Louis, 1 avenue Claude Vellefaux, Paris F75010, France

A second melanoma-susceptibility gene, *CDK4*, was also recently characterized. Two different mutations, both occurring at codon 24 in exon 2 of the gene, were identified in three families, two American and one French (Zuo et al, 1996, Soufir et al, 1998). These mutations were shown to segregate in these CMM families and to prevent $P16^{INK4A}$ binding (Zuo et al, 1996).

Several arguments support that these two genes may play a role in predisposition to UMM. First, UMM cases were present in melanoma families that have been linked to the 9p21 locus (Cannon-Albright et al, 1992). Second, somatic deletions, loss of heterozygosity of 9p21 genetic markers or 5′CpG island methylation of $p16^{INK4A}$ have been detected in UMM cell lines and primary sporadic UMM (Ohta et al, 1994; Speicher et al, 1994; Merbs et al, 1999). Third, a loss of $P16^{INK4A}$-cdk4 proteins interaction was observed in UMM cell lines (Mouriaux et al., 1998). This led us to search for germline mutations in the entire coding sequence of the INK4A-ARF locus (exon 1α, exon 1β, exon 2 and 3) and *CDK4* (exons 2, 5 and 8) in individuals either with a familial history of UMM or with a personal history of melanoma, in addition to UMM.

### PATIENTS AND METHODS

### Families and patients selection

One hundred and fifty-five individuals (56% female, 44% male) affected with uveal melanoma and followed at the Institut Curie between January 1994 and September 1997 were interviewed about their personal and familial history of melanoma. Patients with at least an uveal or cutaneous melanoma in a first- to third-degree relative, or with a personal history of second uveal or cutaneous melanoma were proposed for genetic interview. Further information on family members was therefore collected, including age at diagnoses, site of melanoma tumours and occurrence of other malignancies in affected family members. Diagnosis were based on ocular fundus examination, ultrasound and angiography, and cases treated by enucleation were confirmed by histopathology. Clinical examination of the skin in index cases looked for a dysplastic naevus syndrome (defined by more than 50 naevi, with at least one atypical mole). A blood sample was obtained by venipuncture, with their informed consent.

### Molecular analysis

Genomic DNA was prepared from peripheral blood mononuclear cells isolated by centrifugation through a Ficoll gradient. Each of the four exons of the INK4A-ARF locus and the intron–exon junctions were analysed by polymerase chain reaction single-strand conformation polymorphism (PCR-SSCP). Primer sequences for the exons 1α, 2, and 3 of the INK4A-ARF locus were described in Soufir et al (1998). Exon 1β was analysed through two overlapping PCR products generated by using the following primers: p19F1 5′-TCAGGGAAGGGCGGGTGCG-3′, P19R1 5′-GCC-GCGGGATGTGAACCA-3′ (PCR product: 245 bp), p19F2 5′-GCCGCGAGTGAGGGTTTT-3′, p19R2 5′-CACCGCGGTTAT-CTCCTC-3′ (PCR product 257 bp).

Functional and mutational studies of *CDK4* gene have focused on the regions involved in $P16^{INK4A}$ binding, particularly in domains encompassing the first 58 N-terminal aminoacids or the C-terminal region (Zuo et al, 1996; Coleman et al, 1997; Byeon et al, 1998) and corresponding to exons 2, 5 and 8 of the gene. These three exons were amplified using flanking intronic primers: (i) exon 2 CDK4F2 5′-AGCGACTTTTGGTGATAGGAGT-3′, CDK4R2 5′-GGCTGTCTTTTCCCTTTACTC-3′ (PCR product: 322 bp), (ii) exon 5, CDK4F5 5′-AGAGTGATTGCCCGTAGC-3′, CDK4R5 5′-GCAAGGTATGGATGTGGT-3′ (PCR product: 296 bp), and (iii) exon 8, CDK4F8 5′-GCTCATCCCAGGTATTGT-3′, CDK4R8 5′-TTGCCCTCTCAGTGTCCA-3′ (PCR product: 234 bp). Total PCR reactions volume was 20 μl including 50–100 ng of genomic DNA template, 30 pmol of each primer, 0.5 UI of *Taq* polymerase (Gibco-BRL) and 0.1 μl of α33P-dCTP (Amersham). Final magnesium chloride ($MgCl_2$) concentration was 1.5 mM for all PCR reactions. All reactions were supplemented with 10% dimethyl sulphoxide (DMSO) for optimal PCR amplification. PCR was processed with an annealing temperature of 60°C for the four exons of the INK4A-ARF locus, and 55°C for the three studied exons of *CDK4*. For SSCP, all PCR products were migrated on two 0.1 × TBE/Hydrolink MDE gels (FMC Bioproducts), with 8% glycerol at room temperature (RT) and without glycerol at +4°C. Gels were run at 8W, either 14 h at RT or 12 h at 4°C, dried and exposed for autoradiography. Sample containing $p16^{INK4A}$ exon 3 variant migrating band was sequenced on both strands by automated sequencing using the primers mentioned above. Products from 50-μl PCR reactions were purified using a Microcon 100 (Amicon) and sequenced using dye-labelled terminator on an automated sequencer 373 (Applied Biosystem) according to the manufacturer's instructions.

### RESULTS

### Clinical data

Among the 155 patients with UMM, nine patients (5.8%) were selected for genetic analysis (Table 1). Nineteen out of 20 melanoma cases (95%) could be confirmed from medical records. Seven patients had one relative affected with UMM (families 1–4, 6 and 7) or CMM (family 5). In three families, the affected cases were first-degree related (families 1–3); in two, they were second-degree related (families 4 and 5), and in the last two, they were third-degree related (families 6 and 7). In the families 3 and 7, the affected relative had, in addition to UMM, a CMM. Besides these seven familial cases, two patients reported a history of second primary melanoma (families 8 and 9). One had a bilateral UMM (family 8) and the other, a CMM (family 9). Median age at diagnosis was 56 years old (41–80), and sex ratio, three women for one man. Only one proband (family 9) had a typical dysplastic naevus syndrome, with numerous atypical moles. In three families, different malignancies were also present, all confirmed from medical records (Table 1). In family 3, a prolactin adenoma was observed in both affected relatives, in addition to UMM. In family 5, a bilateral breast cancer was present in the index case, as well as a colon cancer and an ovary cancer in the affected relative. In family 9, the index case had in addition a basal cell carcinoma.

### Molecular analysis

Each proband was screened by PCR-SSCP for germline mutation of the INK4A-ARF locus. No abnormal pattern was detected either in exon 1α or in exon 2. One abnormal migrating pattern was observed in exon 3 in one out of nine samples (family 8),

© 2000 Cancer Research Campaign 

**Table 1** Patients with a family or personal history of uveal melanoma: characteristics of index cases and affected family members selected for this study

| Selected cases | Sex | Tumour type | Age at diagnosis | Affected relatives | Other primary malignancy | DNS |
|---|---|---|---|---|---|---|
| Family 1 | | | | | | |
| Index case | F | **UMM** | 43 | | * | * |
| Affected relative | M | **UMM** | 62 | Father | * | |
| Family 2 | | | | | | |
| Index case | F | **UMM** | 62 | | * | * |
| Affected relative | M | **UMM** | 80 | Father | * | |
| Family 3 | | | | | | |
| Index case | F | **UMM** | 70 | | **Prolactinoma** (60) | * |
| Affected relative | F | **UMM**, **CMM** | 44, 63 | Sister | **Prolactinoma** (59) | |
| Family 4 | | | | | | |
| Index case | F | **UMM** | 48 | | * | * |
| Affected relative | F | **UMM** | 49 | Maternal aunt | * | |
| Family 5 | | | | | | |
| Index case | F | **UMM** | 69 | | **Bilateral breast K** (52, 53) | * |
| Affected relative | F | **CMM** | 42 | Niece | **Colon K (42), ovary K** (43) | |
| Family 6 | | | | | | |
| Index case | F | **UMM** | 33 | | * | * |
| Affected relative | M | **UMM** | 42 | Paternal cousin | * | |
| Family 7 | | | | | | |
| Index case | F | **UMM** | 69 | | * | * |
| Affected relative | M | **UMM**, **CMM** | 67, 63 | Maternal cousin | * | |
| Family 8 | | | | | | |
| Index case | F | **Bilateral UMM** | 69, 69 | * | * | * |
| Family 9 | | | | | | |
| Index case | F | **UMM**, **CMM** | 41, 44 | * | **Basal cell carcinoma** (40) | yes |

DNS, dysplastic naevus syndrome. In **bold** letters, cases whose diagnosis was confirmed by medical records.

corresponding to a previously reported nucleotide variant C to G at position 500 (nucleotide numbering beginning at the first ATG site), located in the *p16*$^{INK4A}$ cDNA 3′ untranslated region (Chaubert et al, 1996). In addition, no sequence variant in exon 1β of the *p14*$^{ARF}$ gene was detected in any of the samples, therefore suggesting that germline mutations in the INK4A-ARF locus are not a common event in genetic predisposition to UMM. SSCP analysis of the functional domains of the *CDK4* gene did not reveal any SSCP variants in the nine samples.

### DISCUSSION

We have investigated nine white French families that had a highly suspected UMM predisposition, owing to a personal or familial history of multiple melanomas. Approximately 1% of patients affected with UMM report a first- to third-degree relative affected with the same disease (Singh et al, 1996*a*). The higher frequency of familial cases observed in our study (3.9%), as compared with previous reports, may reflect (i) sampling variation, or (ii) a slight bias towards the ascertainment of familial cases due to the availability of genetic counselling consultation at the Institut Curie. Nevertheless, our data confirm the association between UMM and CMM, either in relatives of UMM cases, or in the same individuals (families 3, 5, 7 and 9) as previously reported (Bataille et al, 1993).

We did not detect any mutation in the coding sequence of either the INK4A-ARF locus or *CDK4* gene in the nine families studied. We cannot rule out misdetection of mutations by SSCP, but this seems unlikely, as we recently reported one of the highest rates of *p16*$^{INK4A}$ germline mutation in CMM families, using the same technical approaches (Soufir et al, 1998). Our data enhance results of two previous studies: Wang et al did not find any pathogenic mutation in 13 UMM patients with a family history of melanoma, either uveal ($n$ = 6), or cutaneous ($n$ = 7) (Wang et al, 1996); Singh et al did not detect either any mutation in eight families with two members affected with UMM (Singh et al 1996*c*). All 30 UMM families studied worldwide up to now have in common an absence of mutation in *p16*$^{INK4A}$, therefore supporting the rare involvement of this gene in genetic predisposition to UMM.

A single nucleotide substitution (G to C) was detected in the 3′ untranslated region of exon 3 of *p16*$^{INK4A}$, in one out of nine patients (11%), affected with bilateral UMM (family 8). This nucleotide variant has been previously assessed as being a silent polymorphism with a maximum frequency of 25% in a European white population (Chaubert et al, 1996), and of 29% in a set of sporadic CMM (Kumar et al, 1998). However, more recently, Aitken et al reported that in CMM prone-families from Queensland, this polymorphism might be overrepresented as compared to a healthy population and could therefore have a deleterious effect (Aitken et al, 1999). This seems unlikely in UMM given the low frequency of this variant in our series of nine patients.

Liu et al recently reported a germ-line transversion G→T at base –34 of *p16*$^{INK4A}$ (in the 5′ UTR), creating an aberrant initiation codon, in two CMM families (Liu et al, 1999). In our study, we detected no mutation up to 104 bp upstream of the first translation site. Nonetheless, we cannot rule out mutations in the promoter or other regulatory region of *p16*$^{INK4A}$. We did not detect any germline mutation in exon 1β from *p14*$^{ARF}$ in our set of families, which supports that this gene is not involved in predisposition to UMM. Yet, it should be pointed out that large hemizygous deletions of the INK4A-ARF locus may have escaped our mutation detection

 *© 2000 Cancer Research Campaign*

strategy. However, such genomic rearrangements seem rather to predispose to melanoma-astrocytoma syndrome (Bahuau et al, 1998).

We did not find any germline mutation in *CDK4* gene. Thus, despite the fact that our series is small and *CDK4* is less frequently involved in familial CMM than $p16^{INK4A}$, *CDK4* gene does not seem to play a major role in genetic predisposition to UMM. This conclusion is consistent with previous data reporting the absence of somatic mutation of this gene in 30 primary uveal melanomas (Tsao et al, 1998). However, to rule out definitively *CDK4* as a UMM predisposing gene, further studies should be performed on larger family sets.

Genes other than $p16^{INK4A}$, $p14^{ARF}$ and *CDK4* might be involved in predisposition to UMM. First, involvement of another gene localized within the 9p21 locus is supported by (i) the occurrence of somatic deletions of 9p21 markers in up to 30% of UMM tumours (Otah et al, 1994), (ii) the presence of homozygous somatic deletions sparing the INK4A-ARF locus in CMM tumours (Puig et al, 1995), and (iii) the absence of $p16^{INK4A}$ germline mutation in cutaneous melanoma kindreds linked to 9p21 locus (Kamb et al, 1994; Liu et al, 1997). Second, a third melanoma susceptibility locus at 1p36 was initially characterized for the combined melanoma/dysplastic naevus trait (Bale et al, 1989; Goldstein et al, 1993). As UMM and CMM may be genetically related via the sporadic DNS or the FAMMM syndrome (Bataille et al, 1993; Singh et al, 1996*c*; Van Hees et al, 1998), this locus might be involved in genetic predisposition to UMM. Third, *BRCA2* germline mutations might account for some UMM cases associated with a personal history of breast cancer (Sinilnikova et al, 1999). However, in family 7, no germline BRCA2 mutation was detected despite the strong suggestion for the involvement of this gene (Table 1) (Sinilnikova et al, 1999).

Finally, loss of heterozygosity and cytogenetic studies have suggested that tumour suppressor genes on chromosomes 2, 3 and 6, as well as an oncogene on chromosome 8q, could be involved in the tumorigenic process of uveal tumours (Prescher et al, 1994).

In conclusion, our results suggest that germline mutations of $p16^{INK4A}$, $p14^{ARF}$ and *CDK4* genes are not frequently observed in genetic predisposition to UMM. This confirms genetic heterogeneity, therefore supporting the existence of additional melanoma susceptibility genes.

### ACKNOWLEDGEMENTS

This work was supported by grants from the Ligue contre le Cancer des Yvelines and the AP-HP (CRC96011). Nadem Soufir is a recipient of a fellowship from the Ligue Nationale contre le Cancer. We thank Dr Agnes Chompret for useful discussion, Isabelle Eugène for the management of family data and Anne de Henning for the linguistic revision of the manuscript.

### REFERENCES

Aitken J, Welch J, Duffy D, Milligan A, Green A, Martin N and Hayward N (1999) CDKN2A variants in a population-based sample of Queensland families with melanoma. *J Natl Cancer Inst* **91**: 446–452

Bale SJ, Dracopoli NC, Tucker MA, Clark WH Jr, Fraser MC, Stanger BZ, Green P, Donis-Keller H, Housman DE and Greene MH (1989) Mapping the gene for hereditary cutaneous malignant melanoma-dysplastic nevus to chromosome 1p. *N Engl J Med* **320**: 1367–1372

Bahuau M, Vidaud D, Jenkins RB, Bieche I, Kimmel DW, Assouline B, Smith JS, Alderete B, Cayuela JM, Harpey JP, Caille B and Vidaud M (1998) Germ-line deletion involving the INK4 locus in familial proneness to melanoma and nervous system tumours. *Cancer Res* **58**: 2298–3003

Bataille V, Pinney E, Hungerford JL, Cuzick J, Bishop DT and Newton JA (1993) Five cases of coexistent primary ocular and cutaneous melanoma. *Arch Dermatol* **129**: 198–201

Byeon IJ, Li J, Ericson K, Selby TL, Tevelev A, Kim HJ, O'Maille P and Tsai MD (1998) Tumour suppressor p16INK4A: determination of solution structure and analyses of its interaction with cyclin-dependent kinase 4. *Mol Cell* **1**: 421–431

Canning CR and Hungerford J (1988) Familial uveal melanoma. *Br J Ophthalmol* **72**: 241–243

Cannon-Albright LA, Goldgar DE, Meyer LJ, Lewis CM, Anderson DE, Fountain JW, Hegi ME, Wiseman RW, Petty EM, Bale AE, Olopade OI, Diaz MO, Kwiatkowski DJ, Piepkorn MW, Zone JJ and Skolnick MH (1992) Assignment of a locus for familial melanoma, MLM, to chromosome 9p13–p22. *Science* **258**: 1148–1152

Chaubert P, Shaw P and Pillet N (1996) Informative MspI polymorphism adjacent to exon 3 of the p16INK4 (MTS1) gene. *Mol Cell Probes* **10**: 467–468

Coleman KG, Wautlet BS, Morrissey D, Mulheron J, Sedman SA, Brinkley P, Price S and Webster K (1997) Identification of CDK4 sequences involved in cyclin D1 and p16 binding. *J Biol Chem* **272**: 18869–18874

Dracopoli NC and Fountain JW (1996) CDKN2 mutations in melanoma. *Cancer Surv* **26**: 115–132

Easton DF, Steele L, Fields P, Ormiston W, Averill D, Daly PA, McManus R, Neuhausen SL, Ford D, Wooster R, Cannon-Albright LA, Stratton MR and Goldgar D (1997) Cancer risks in two large breast cancer families linked to BRCA2 on chromosome 13q12–13. *Am J Hum Genet* **61**: 120–128

Goldstein AM, Dracopoli NC, Ho EC, Fraser MC, Kearns KS, Bale SJ, McBride OW, Clark WH Jr and Tucker MA (1993) Further evidence for a locus for cutaneous malignant melanoma-dysplastic nevus (CMM/DN) on chromosome 1p, and evidence for genetic heterogeneity. *Am J Hum Genet* **52**: 537–550

Kamb A, Shattuck-Eidens D, Eeles R, Liu Q, Gruis NA, Ding W, Hussey C, Tran T, Miki Y, Weaver-Feldhaus J, McClure M, Aitken JF, Anderson DE, Bergman, W, Frants R, Goldgar DE, Green A, MacLennan R, Martin NG, Meyer LJ, Youl P, Zone JJ, Skolnick MH and Cannon-Albright LA (1994) Analysis of the p16 gene (*CDKN2*) as a candidate for the chromosome 9p melanoma susceptibility locus. *Nat Genet* **8**: 22–26

Kamijo T, Weber JD, Zambetti G, Zindy F, Roussel MF and Sherr CJ (1998) Functional and physical interactions of the ARF tumour suppressor with p53 and Mdm2. *Proc Natl Acad Sci USA* **95**: 8292–8297

Kumar R, Sauroja I, Punnonen K, Jansen C and Hemminki K (1998) Selective deletion of exon 1beta of the p19ARF gene in metastatic melanoma cell lines. *Genes Chromosomes Cancer* **23**: 273–277

Liu L, Goldstein AM, Tucker MA, Brill H, Gruis NA, Hogg D and Lassam NJ (1997) Affected members of melanoma-prone families with linkage to 9p21 but lacking mutations in CDKN2A do not harbor mutations in the coding regions of either CDKN2B or p19ARF. *Genes Chromosomes Cancer* **19**: 52–54

Liu L, Dilworth D, Gao L, Monzon J, Summers A, Lassam N and Hogg D (1999) Mutation of the CDKN2A 5′ UTR creates an aberrant initiation codon and predisposes to melanoma. *Nat Genet* **21**: 128–132

Lynch HT, Anderson DE and Krush AJ (1968) Heredity and intraocular malignant melanoma. Study of two families and review of forty-five cases. *Cancer* **21**: 119–125

Merbs SL and Sidransky D (1999) Analysis of p16 (CDKN2/MTS-1/INK4A) alterations in primary sporadic uveal melanoma. *Invest Ophthalmol Vis Sci* **40**: 779–783

Mouriaux F, Casagrande F, Pillaire MJ, Manenti S, Malecaze F and Darbon JM (1998) Differential expression of G1 cyclins and cyclin-dependent kinase inhibitors in normal and transformed melanocytes. *Invest Ophthalmol Vis Sci* **39**: 876–884

Newton Bishop JA, Bataille V, Pinney E and Bishop DT (1998) Family studies in melanoma: identification of the atypical mole syndrome (AMS) phenotype (1994). *Melanoma Res* **4**: 199–206

Ohta M, Nagai H, Shimizu M, Rasio D, Berd D, Mastrangelo M, Singh AD, Shields JA, Shields CL, Croce CM, et al (1994) Rarity of somatic and germ-line mutations of the cyclin-dependent kinase 4 inhibitor gene, CDK4I, in melanoma. *Cancer Res* **54**: 5269–5272

Prescher G, Bornfeld N and Becher R (1994) Two subclones in a case of uveal melanoma: relevance of monosomy 3 and multiplication of chromosome 8q. *Cancer Genet Cytogenet* **77**: 144–146

Puig S, Ruiz A, Lazaro C, Castel T, Lynch M, Palou J, Vilalta A, Weissenbach J, Mascaro JM and Estivill X (1995) Chromosome 9p deletions in cutaneous malignant melanoma tumours: the minimal deleted region involves markers outside the p16 (CDKN2) gene. *Am J Hum Genet* **57**: 395–402

© 2000 Cancer Research Campaign 

Serrano M, Hannon GJ and Beach D (1993) A new regulatory motif in cell-cycle control causing specific inhibition of cyclin D/CDK4. *Nature* **366**: 704–707

Singh AD, Wang MX, Donoso LA, Shields CL, Potter PD, Shields JA, Elston RC and Fijal B (1996*a*) Familial uveal melanoma, III. Is the occurrence of familial uveal melanoma coincidental? *Arch Ophthalmol* **114**: 1101–1104

Singh AD, Shields CL, De Potter P, Shields JA, Trock B, Cater J and Pastore D (1996*b*) Familial uveal melanoma. Clinical observations on 56 patients. *Arch Ophthalmol* **114**: 392–399

Singh AD, Croce CM, Wary KK, Shields JA, Donoso LA, Shields CL, Huebner K and Ohta M (1996*c*) Familial uveal melanoma: absence of germ-line mutations involving the cyclin-dependent kinase-4 inhibitor gene (p16). *Ophthalmic Genet* **17**: 39–40

Singh AD, Shields CL, Shields JA and De Potter P (1996*d*) Bilateral primary uveal melanoma. Bad luck or bad genes? *Ophthalmology* **103**: 256–262

Singh AD, Wang MX, Donoso LA, Shields CL, De Potter P and Shields JA (1996*e*) Genetic aspects of uveal melanoma: a brief review. *Semin Oncol* **23**: 768–772

Sinilnikova OM, Egan KM, Quinn JL, Boutrand L, Lenoir GM, Stoppa-Lyonnet D, Desjardins L, Levy C, Goldgar D and Gragoudas ES (1999) Germ-line BRCA2 sequence variants in patients with ocular melanoma. *Int J Cancer* **82**: 323–328

Speicher MR, Prescher G, du Manoir S, Jauch A, Horsthemke B, Bornfeld N, Becher R and Cremer T (1994) Chromosomal gains and losses in uveal melanomas detected by comparative genomic hybridization. *Cancer Res* **54**: 3817–3823

Soufir N, Avril MF, Chompret A, Demenais F, Bombled J, Spatz A, Stoppa-Lyonnet D, Benard J and Bressac-de Paillerets B (1998) Prevalence of p16 and CDK4 germ-line mutations in 48 melanoma-prone families in France. The French Familial Melanoma Study Group. *Hum Mol Genet* **7**: 209–216

Stott FJ, Bates S, James MC, McConnell BB, Starborg M, Brookes S, Palmero I, Ryan K, Hara E, Vousden KH and Peters G (1998) The alternative product from the human CDKN2A locus, p14(ARF), participates in a regulatory feedback loop with p53 and MDM2. *EMBO J* **17**: 5001–5014

Tsao H, Benoit E, Sober AJ, Thiele C and Haluska FG (1998) Novel mutations in the p16/CDKN2A binding region of the cyclin-dependent kinase-4 gene. *Cancer Res* **58**: 109–113

Van Hees CL, Jager MJ, Bleeker JC, Kemme H and Bergman W (1998) Occurrence of cutaneous and uveal melanoma in patients with uveal melanoma and their first-degree relatives. *Melanoma Res* **8**: 175–180

Wang X, Egan KM, Gragoudas ES and Kelsey KT (1996) Constitutional alterations in p16 in patients with uveal melanoma. *Melanoma Res* **6**: 405–410

Zuo L, Weger J, Yang Q, Goldstein AM, Tucker MA, Walker GJ, Hayward N and Dracopoli NC (1996) Germ-line mutations in the P16INK4a binding domain of CDK4 in familial melanoma. *Nat Genet* **12**: 97–99

*© 2000 Cancer Research Campaign*
